# Development and Study of Semi-Solid Preparations Containing the Model Substance Corticotropin (ACTH): Convenience Application in Neurodegenerative Diseases

**DOI:** 10.3390/molecules25081824

**Published:** 2020-04-16

**Authors:** Wioletta Siemiradzka, Barbara Dolińska, Florian Ryszka

**Affiliations:** 1 Department of Pharmaceutical Technology, School of Pharmacy with the Division of Laboratory Medicine in Sosnowiec, Medical University of Silesia in Katowice, 41-200 Sosnowiec, Poland; bdolinska@sum.edu.pl; 2 “Biochefa” Pharmaceutical Research and Production Plant, 41-200 Sosnowiec, Poland; f.ryszka@biochefa.pl

**Keywords:** semi-solid dosage forms, ACTH, physicochemical properties, texture analysis, in vitro drug release study

## Abstract

Corticotropin (ACTH, previously an adrenocorticotropic hormone) is used in the diagnosis and treatment of pituitary gland disorders, adrenal cortex disorders, and other diseases, including autoimmune polymyositis, systemic lupus erythematosus, rheumatoid arthritis, Crohn’s disease, and ulcerative colitis. So far, the ointment dosage form containing ACTH for use on the skin is unknown. Therefore, it seems appropriate to develop a semi-solid formulation with corticotropin. Emulsion ointments were prepared using an Unguator based on the cream base Lekobaza^®^ containing corticotropin in different concentrations, and then the physical and chemical parameters of the ointment formulations, such as pH, spreadability, rheological properties, and texture analysis, were evaluated. In addition, a USP apparatus 2 with enhancer cells was utilized to study the in vitro drug release characteristics of the selected formulations. All the ointments obtained were characterized by good spreadability and viscosity. An analysis of the ointment texture was performed and the dependence of the tested parameters on the ACTH content in the ointment was demonstrated. Examination of the structure of the ointment showed that a high concentration of ACTH increases the hardness and adhesiveness of the ointment. In turn, it adversely affects the cohesiveness and elasticity of the ointments tested. The results of the release study showed that ACTH is released the fastest from the formulation with the lowest concentration, while the slowest from the ointment with the highest concentration of ACTH.

## 1. Introduction

Currently, about 74% of drugs taken orally do not provide the expected therapeutic effect. In order to improve the effectiveness of therapy, much attention is devoted to the development of safe and effective ways of delivering drugs through the skin due to the continuous development of new and innovative approaches [1]. In addition to its protective function, the skin, thanks to its large surface area of approximately 1–2 m^2^ (about 15% of the total body weight) [2,3] and good blood supply to the dermis (skin microcirculation) [4,5,6], can be perfectly used for the administration of therapeutic substances, both topical and systemic. This non-invasive and convenient route of administration (transdermal route) seems to be a promising way of overcoming difficulties related to the use of peptide substances [1,2,4,7,8,9,10]. The ability of approved transdermal drugs to penetrate the skin varies widely and it depends on the physicochemical properties of drug molecules. There are two possible routes of drug penetration across the intact skin: The transepidermal and transappendegeal pathways. The transepidermal pathway involves the passage of molecules through the stratum corneum. Transepidermal penetration can be named intra- or inter-cellular [4,11]. The intra-cellular route through corneocytes allows the transport of hydrophilic or polar molecules. Transport via inter-cellular spaces allows the diffusion of lipophilic or non-polar molecules through the continuous lipid layer. The transappendegeal route involves the passage of molecules through sweat glands and across the hair follicles [4,8,12].

Percutaneous absorption of molecules is a step process involving the penetration of a substance into a particular layer of the skin, entrance from the stratum corneum into the aqueous viable epidermis, and diffusion through the viable epidermis and into the upper dermis, permeation: The penetration of molecules from one layer into another and the uptake of a substance into the systemic circulation.

Based on Fick’s law of diffusion, the transport of therapeutic molecules across skin will be maintained until the concentration gradient ceases to exist [4,13,14,15]. Transdermal systems should be formulated to provide the maximum thermodynamic driving force for passive diffusion across the skin, which is saturated with a sufficient payload of the drug to ensure delivery of drugs across the skin.

Corticotropin (ACTH) is a peptide hormone of the anterior pituitary gland consisting of 39 residual amino acids with a molecular weight of 4.5 kDa [16,17,18] and biological activity of 100 IU/mg. In the organism, ACTH is characterized by a short half-life of t_0.5_, ranging from 5 to 15 min (due to proteolytic degradation) [18]. The correct range of plasma ACTH concentrations is 1.5–11 pmol/L for men and 1.1–5.9 pmol/L for women [19]. 

ACTH is used in the diagnostics and treatment of pituitary-adrenal cortex diseases and other disorders, including autoimmune polymyositis, systemic lupus, rheumatoid arthritis, Crohn’s disease, and ulcerative colitis, as well as inflammatory and allergic processes of the eye [20,21]. Three preparations containing ACTH are registered and used: H.P. Acthar^®^ Gel, Cortrosyn TM, and Synacthen^®^ Depot [21,22]. All these preparations are injected. H.P. Acthar^®^ Gel is an injectable form for intramuscular or subcutaneous administration containing 80 IU/mL (0.4 mg/mL) of natural ACTH [20,23,24].

Injection to a patient in long-term therapy is quite uncomfortable and invasive. So far, the literature lacks reports of a semi-solid form containing ACTH for skin application. The application of the drug topically on the skin or mucous membranes has many advantages. It allows passage through the gastrointestinal tract to be avoided and thus the effect of the first pass effect. The effect of the drug does not depend on pH or the degree of stomach filling. Easy application, non-invasiveness, and painlessness of using ensure acceptance by the patient. These forms of the drug may have a protective effect on the skin, thanks to which it is possible to achieve a protective effect in case of pathologically changed skin. They are referred to in hydrophilic creams, such as Lekobaza^®^, which has been applied as a base in tests for the preparation of an ointment with corticotropin. Lekobaza^®^ contains glycerol monostearate, cetyl alcohol, medium chain triglycerides, white petrolatum, macrogol-20-glycerol monostearate, propylene glycol, and 40% water. It does not contain preservatives, and microbiological protection in this medium is provided by 20% propylene glycol per aqueous phase. It spreads well and is washed off with water. The composition of the substrate and properties of the active substance should ensure, among others, easy spreadability on the skin and good adhesion to it. These characteristics result from the consistency of the ointment.

The aim of the study was to develop a new semi-solid form of a drug containing corticotropin with appropriate physicochemical properties, allowing the active substance to penetrate the skin barrier and reach a therapeutic concentration, thus showing effective action. In the ointment, the therapeutic concentration depends on the surface of the ointment applied to the skin and the amount released, which penetrates the skin. Assuming that the availability of therapeutic substances from the ointment is about 10%–20%, it can be expected that the therapeutic range of ACTH will be in the range from 1.5 to 5.0 mg (for 10% and 20% availability for concentrations from 15 to 25 mg/g). This range is provided by dosing an ointment of 1.0 g per skin surface. Therefore, the aim of the study was to prepare ointments containing different corticotropin concentrations to determine the physicochemical parameters of the ointment, to test the release of ACTH from the ointment, and to assess the effect of the applied concentration on the tested properties.

## 2. Results and Discussion

### 2.1. Ointment pH Measurements by the Potentiometric Method

Table 1 shows the results of the pH measurements. Measurements were taken for Lekobaza^®^ (F-1) itself, for Lekobaza^®^ with the addition of 1 mol/L aqueous acetic acid solution (F-2), and for ointments with corticotropin with the following concentrations: 5 mg ACTH/g ointment (F-3), 10 mg/g (F-4), 15 mg/g (F-5), 20 mg/g (F-6), and 25 mg/g (F-7). 

The highest value of pH (6.30) was noted for Lekobaza^®^. The addition of 1 mol/L acetic acid solution significantly reduced the pH to 3.43. Corticotropin added to the ointment, depending on its concentration, caused in turn a slight increase in the pH value in comparison with the ointment with the 1 mol/L acetic acid solution only. The more corticotropin in the ointment, the higher the pH value. The lowest pH value was the ointment with ACTH 5 mg/g (pH = 3.61), and the highest pH value was the ointment with ACTH 25 mg/g (pH = 4.00). Some excipients may increase the pH of the skin, causing skin barrier damage [25], while other ingredients may have a beneficial effect by lowering the pH of the skin [26] and protecting the acidic skin coat. For example, the inborn antimicrobial properties of the skin are optimal for acidic pH, because the *Staphylococcus* and other pathogenic bacteria promote a neutral pH and are inhibited in an acidic environment [27]. Moreover, in an acidic environment, the correct exfoliation of the stratum corneum is a process regulated by the kallikrein enzymes “KLK5” and “KLK7” [28]. However, at higher pH, skin cell flaking may get out of control, damaging the stratum corneum barrier [25,26,27,28,29]. 

Corticotropin is an alkaline substance. In an acidic environment, such as a 1 mol/L acetic acid solution, it is possible to increase the stability of this peptide, which remains in the salt solution, acetate. ACTH is dissolved in 0.1 mol/L acetic acid, but acetic acid of 1 mol/L was used to enhance this effect. During corticotropin extraction from the pituitary glands, the extraction takes place in an acidic environment, in order to make the penetration of this hormone into the solvent more effective. The pH value was determined in the tested ointments, and no pH modification was introduced here yet, as these were preliminary studies. However, this modification requires testing of the stability of ACTH by increasing the pH.

### 2.2. Spreadability of the Ointments

**Figure 1:**
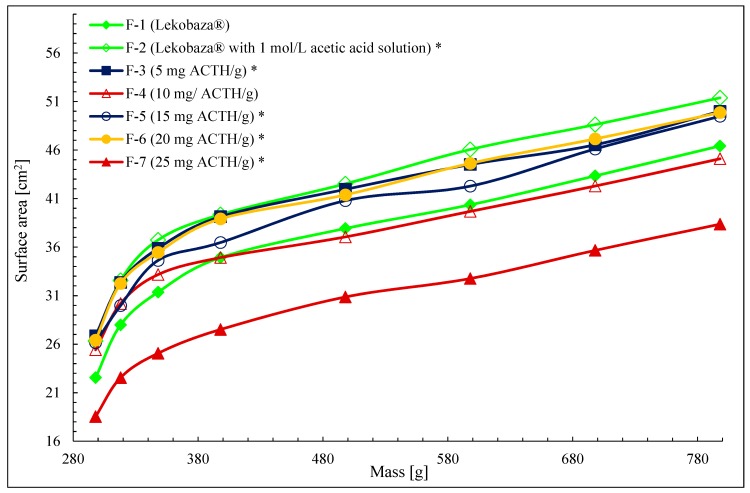
The dependence of the sample surface area vs. applied mass, *—the statistically significant difference relates to F-1 (*p* < 0.05).

In order to evaluate the effect of the addition of an aqueous solution, two formulations were compared. One formulation was a control and it was Lekobaza^®^ only, and the second formulation was Lekobaza^®^ with the addition of a 10% aqueous acetic acid solution of 1 mol/L. In order to present the effect of the ACTH content on the ointment spreadability, the course of spreadability curves were compared with this parameter for the control ointment with the acetic acid solution (F-2). The course of the spreadability curves is shown in Figure 1.

Lekobaza^®^ is a base with good spreadability in the assessment of the manufacturer (Fagron, Kraków, Poland). The assessment of the spreadability of a topical or mucosal semi-solid preparation is influenced by the hardness or hardness of the composition, shear rate and time produced after smearing, and temperature at the target site. The spreading efficiency also depends on the formulation viscosity, solvent evaporation rate, and evaporation rate and intensity, with an increase in viscosity along with evaporation. 

The addition of 10% aqueous solution to Lekobaza^®^ (F-1) makes the spreadability of Lekobaza^®^ with acetic acid solution (F-2) even higher (*p* < 0.05). However, with an increase in the concentration of ACTH introduced into Lekobaza^®^ as a solution in 1 mol/L acetic acid, the spreadability (*p* < 0.05) decreased in comparison with the F-2 preparation (Lekobaza^®^ with the addition of 10% aqueous solution). It proves that with an increasing concentration of ACTH, the spreadability will deteriorate.

It was observed that the ointment with the highest ACTH content (25 mg/g) showed the lowest spreadability and the ointment with the lowest ACTH concentration (5 mg/g) showed the best spreadability. Ointments with concentrations of 10 and 15 mg/g, F-4 and F-5, showed worse spreadability than F-3 (5 mg/g) but better than F-7 (25 mg/g). The exception is a formulation with a concentration of 20 mg/g (F-6). 

The highest concentration of ACTH, 25 mg/g, reduces the spreadability to the greatest extent, followed by 10, 15, 20, and 5 mg/g (*p* < 0.05). The spreadability of the 10 mg/g ACTH ointment did not differ significantly from this parameter for the control ointment (F-1). Table 2 shows the parameters describing the ointment spreadability, including equations describing the course of the spreadability curves, correlation coefficients (R^2^), area under the spreadability curves, and the spreadability index (i(S)) in relation to the control ointment F-1. It was found that when i(S) is greater than 1.0, the tested ointment has better spreadability, and when it is less than 1.0, the ointment has worse spreadability in relation to the F-1 formulation.

Therefore, only ointments F-7 and F-4 have weaker spreadability in relation to F-1. If one assumes that Lekobaza^®^ has good spreadability, ointments with ACTH also show proper spreadability: F-3, F-5, and F-6. This is compensated by the addition of a water solution. For the tested ointments, the area under the spread curve (AUC) was calculated, and a weak negative correlation was found for the area under the curve (AUC [cm^2^]), depending on the ACTH concentration in the ointment (R^2^ = −0.428). Similar results were obtained by Szulc-Musioł et al., who found an inverse correlation between the spreading rate and quercetin content (R^2^ = −0.283, *p* ≤ 0.05) [30].

The rheological features of the ointment—plasticity, flow properties, thixotropy—enable the preparation, spreading, and packing of the ointment. Change of viscosity by shear stress allows spreading of the ointment on the skin, and prevents the ointment from flowing out of the skin. Pressure on the ointment tube creates a certain shear stress, which causes the system to flow. It allows the ointment to be squeezed out of the tube. The appropriate flow limit prevents the ointment from accidentally flowing out. When the ointment is mixed (as well as during heating), its organized internal structure is disturbed. The structure can be restored to its original state by rebuilding it in order to maintain the physical stability of the system. 

In this work, it was expected that the addition of the drug would not deteriorate the spreadability parameter in relation to the base itself, which is characterized by good spreadability and an appropriate consistency. After application of the preparation on the skin, the drug will last for a certain period of time and will ensure the longest possible contact with the drugs without the risk of the preparation flowing out.

### 2.3. Rheological Properties

Viscosity is not a constant value and depends on factors, such as the temperature and shear rate. The storage and application temperature of the preparation can be used for measurements. The range of shear rate can also be selected based on the storage of the ointment or the technique of spreading it on the skin. The rheological tests in references were carried out at 20–37 °C. In our study, the viscosity, flow step test, and flow test were tested at a controlled shear rate at two temperatures, 25 and 32 °C. The resting body temperature is approximately 37 °C and the weighted average skin surface temperature is between 32 and 34 °C. This temperature corresponds to the application of ointment on the skin surface. The temperature of 25 °C corresponds to the room temperature at which the ointments are normally stored, just before its application to the skin. Three shearing rates: 300, 700 and 1100 s^−1^ were used in the tests. The determined values of viscosity and shear stress are presented in Table 3.

The temperature and shear rate significantly affect the viscosity of the formulation. At shear rates of 300 and 700 s^−1^ at 32 °C for formulations without active substance and containing low concentrations of ACTH (5 and 10 mg/g), the viscosity is reduced 2 to 2.5 times. For higher concentrations of ACTH, on the other hand, viscosity increases slightly, about 1.1 times under the same conditions. At the wall speed of 1100 s^−1^ with a temperature increase, viscosity decreases about 1.5 times for F-1–F-5 formulations, while for F-6 and F-7, it practically does not change, and is not statistically significant. Decreasing the viscosity of the formulation during spreading of the preparation on the skin may contribute to the increase of the active substance’s release from the ointment.

The addition of 10% aqueous solution to Lekobaza^®^ in a weight ratio significantly decreased the viscosity, 1.2 times for almost all preparations to 2 times for F-1 (control ointment). This may result from thinning of the base as an emulsion system is formed.

The active substance added to the ointment increased the viscosity depending on the hormone concentration in the ointment. This relation is best visible in comparison with hydrated Lekobaza^®^ F-2. The smallest increase in viscosity was observed at a concentration of 5 mg/g ACTH at 25 °C and it increases as follows: F-2 < F-3 < F-4 < F-5 < F-7 < F-6. The smallest increase in viscosity was observed at a concentration of 10 mg/g ACTH at 32 °C and it increases as follows: F-4 < F-5 < F-7 < F-6. The preparation at a concentration of 20 mg/g ACTH caused the highest increase in viscosity.

To prevent excessive viscosity increases at higher corticotropin concentrations, ACTH is included as an aqueous solution to create an emulsion instead of a suspension ointment. It is possible to improve the physicochemical properties of the formulations, including their spreadability, pharmaceutical availability, and bioavailability, and thus improve the effectiveness of the semi-solid drug forms. In the case of the cream base Lekobaza^®^, it is possible due to its ability to absorb a significant amount of water (water number > 300). Kolpakova et al. (2019) tested ointments, such as emulsions, with a water-soluble protein-polysaccharide complex. It was found that all the tested ointments were visco-structural systems, and the ointment composition, complex concentration, and character of the emulsifier appropriately selected by the formulator provided the necessary thixotropic properties [31].

The rheological parameters of the ointment with different concentrations of ACTH were examined in the study. The hydrophilic medium of a cream character, Lekobaza^®^, was used as an ointment base. ACTH was introduced into the ointment as an aqueous solution in acetic acid. It could be expected that the aqueous solution would decrease in the ointment viscosity and thus increase its spreadability. However, with the increase in the ACTH concentration in the ointment, an increase in viscosity was observed respectively, with the exception of the 20mg/g concentration. An increase in the ACTH concentration from 0.5% to 2.5% for the two shear rates is accompanied by an increase in viscosity. An exception is the ointment with a concentration of 2%, and the causes of this phenomenon may be different. Corticotropin is alkaline in nature and may react with acidic components of the base; perhaps at a concentration of 2%, the effects are more intense. It can also be affected by a charge coming from one of the base components.

**Figure 2:**
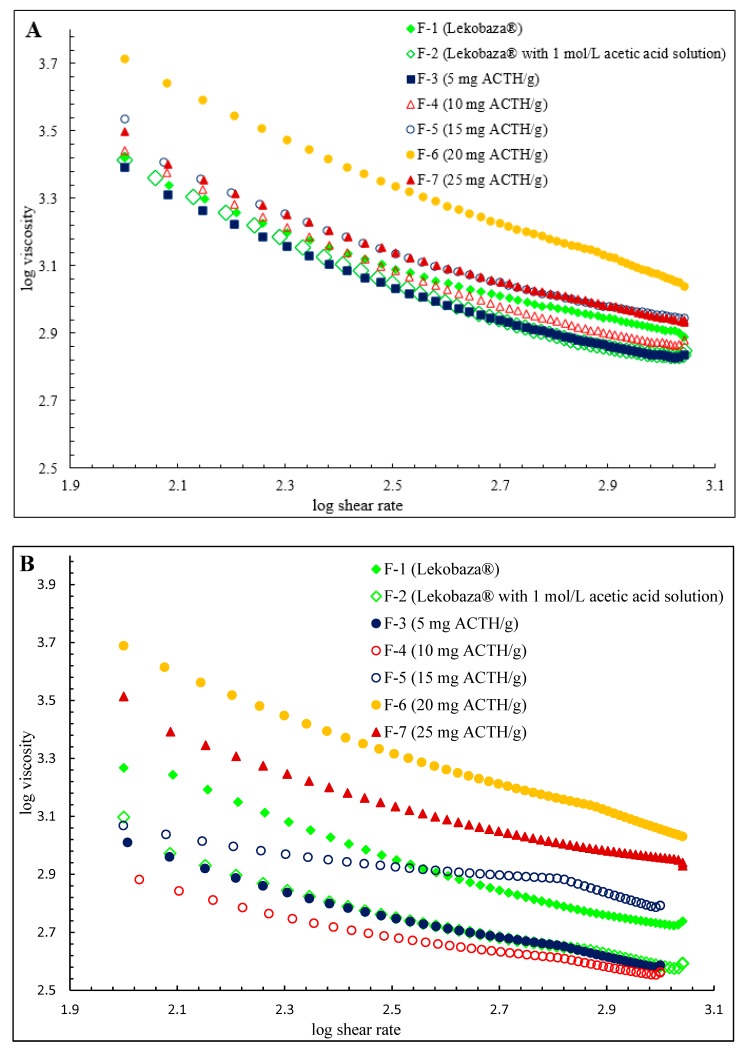
The course of viscosity curves in the controlled shear stress mode as a logarithmic function at 25 (**A**) and 32 °C (**B**).

Lekobaza^®^ contains cetyl alcohol, which also increases the melting point of some ointment bases and suppository media, so it may increase the viscosity of the preparations. Cetyl alcohol also acts as a non-ionic water/oil emulsifier in emulsions. The tested ointments showed a weak negative correlation between the spreadability and viscosity (R^2^ = −0.293 at 25 °C and R^2^ = −0.281 at 32 °C). In turn, Vennat, Gross, and Pourrat, showed the existence of a strong negative correlation between the spreadability and viscosity of elastomeric materials [32,33]. The tested ointments are diluted shear systems. The formulations are shear thinning fluids, and their properties are confirmed by the shape of the viscosity curves in Figure 2A,B. Such properties of Lekobaza^®^ at 25 °C and Lekobaza^®^ with a 20% water content at 32 °C were also demonstrated by Szulc-Musioł et al. and Tal-Figiel et al. [30,34]. 

**Figure 3:**
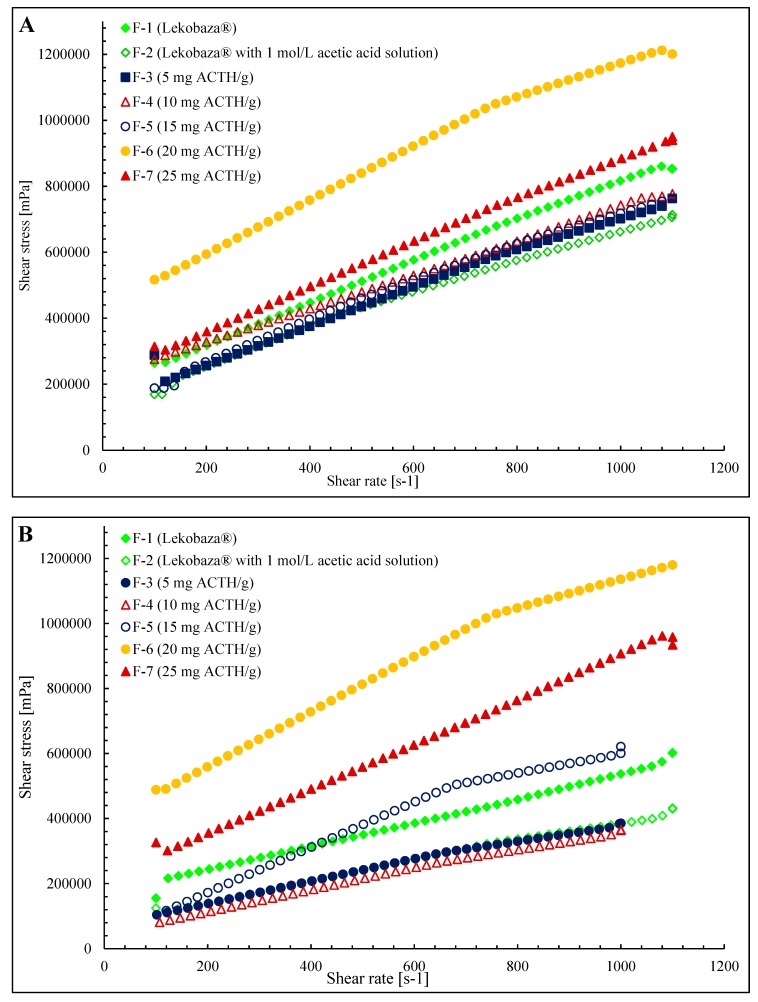
The flow curves of the investigated ointment formulations F-1–F-7 at 25 (**A**) and 32 °C (**B**).

The relationship between the shear stress and shear rate is presented in Figure 3A,B. The course of the flow curves of all prepared ointments indicated a non-Newtonian character. The relationship between shear stress and shear velocity deviated from a straight line, and there was also an offset along the ordinate in the shear rate range from 100 to 1100 s^−1^. Therefore, the equation for non-linear viscoelastic bodies, Casson’s Equation (1), was used to approximate the values of the shear stress and shear rate:

(1) $$\sqrt{\tau }=\sqrt{\tau_{y}}+\sqrt{\eta \gamma },$$

where *τ* = shear stress, *τ_y_* = constant interpreted as yield strength, *η* = constant, and *γ* = strain rate (shear rate). This method allows the determination of the flow limit, and its existence is indicated by the shape of the flow curve [35].

The viscosity of a thixotropic system is dependent on both the shear rate and time. During the process, the structure of the viscoelastic body can be destroyed, and thus its viscosity can be reduced, and its structure can be restored, and its viscosity can increase. Both of these phenomena are significant both from the point of view of the technological process and the application of the finished product to the place of action, the skin. During preparation and mixing, the ointment must lower its viscosity whereas a heightened viscosity is more appropriate for storage or application. High viscosity prevents sedimentation of undissolved particles in the base and movement of the ointment on the skin surface. This would make it difficult for the preparation to act in a specific place (depth of penetration or transport through skin differs due to the thickness of various regions of the skin).

The most suitable method of thixotropy measurement is to describe the material response in shear stress caused by the given deformation or a shear rate [35]. The shear rate increased with time until the maximum shear value was reached. This process is then reversed by decreasing the shear rate, leading to the up/down curves. The area enclosed by the up/down curve is called the hysteresis loop. In these studies, all formulations show thixotropic properties. This is evidenced by the shape of the loop, as the ascending curve is above the descending curve [35]. 

**Figure 4:**
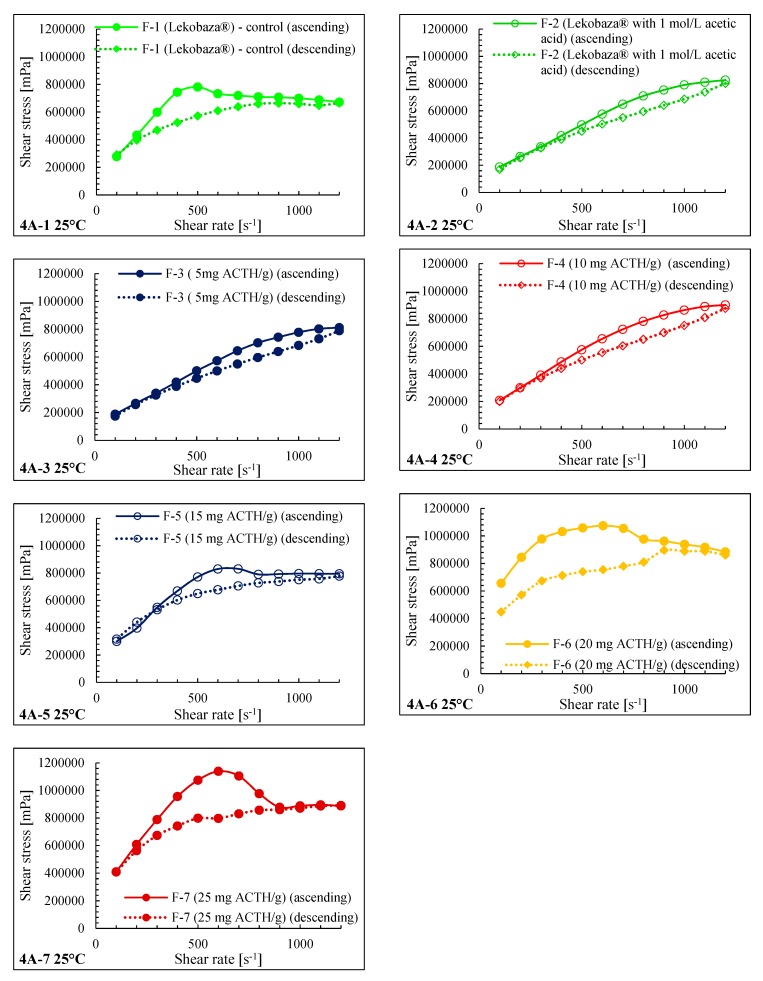

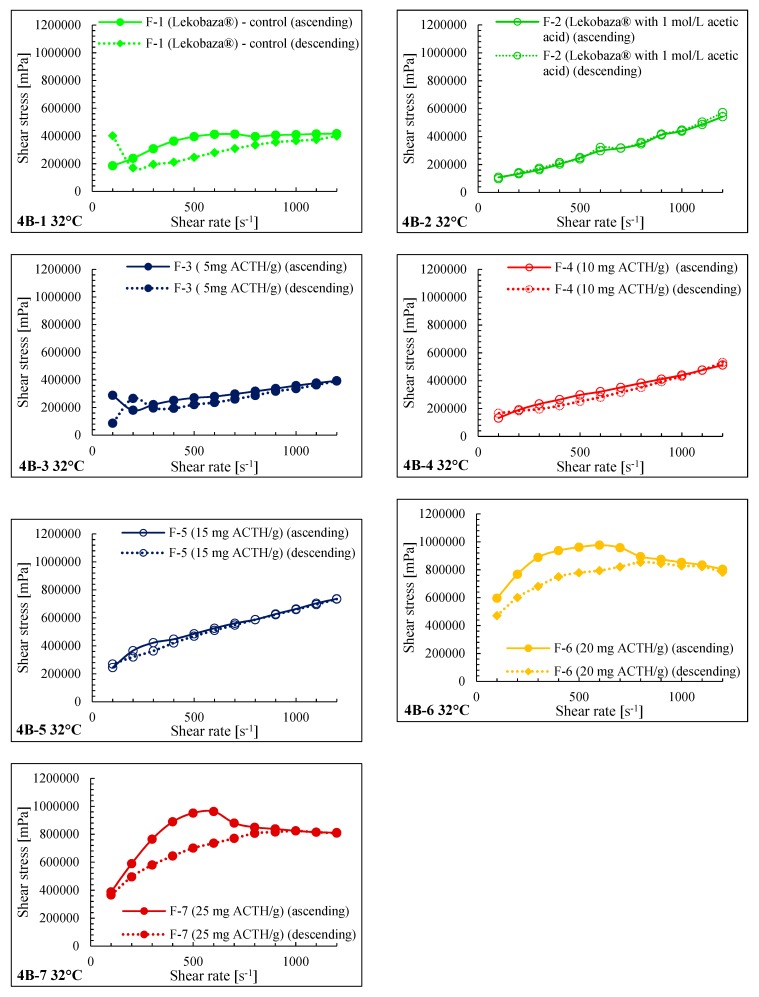

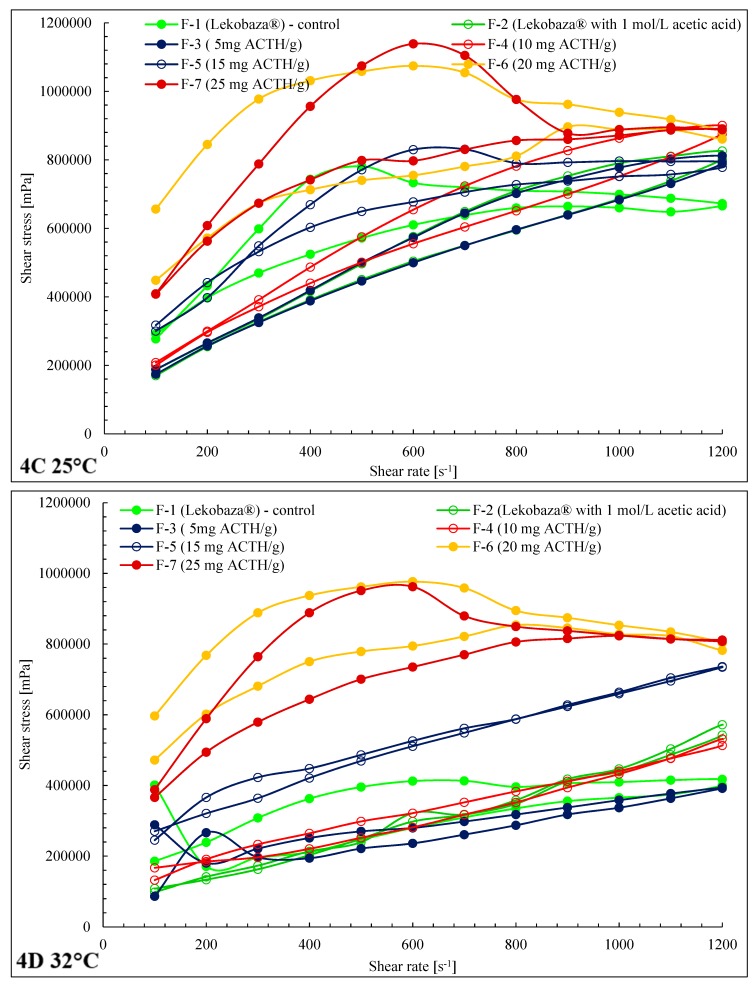
Hysteretic loops for each individual F-2–F-7 and F-1 (reference) formulation of ointment at 25 °C—(**4A-1**)–(**4A-7**), at 32 °C—(**4B-1**)–(**4B-7**) and for all F-2–F-7 and F-1 (reference) simultaneously, at 25 °C (**4C**) and 32 °C—(**4D**).

**Figure 5:**
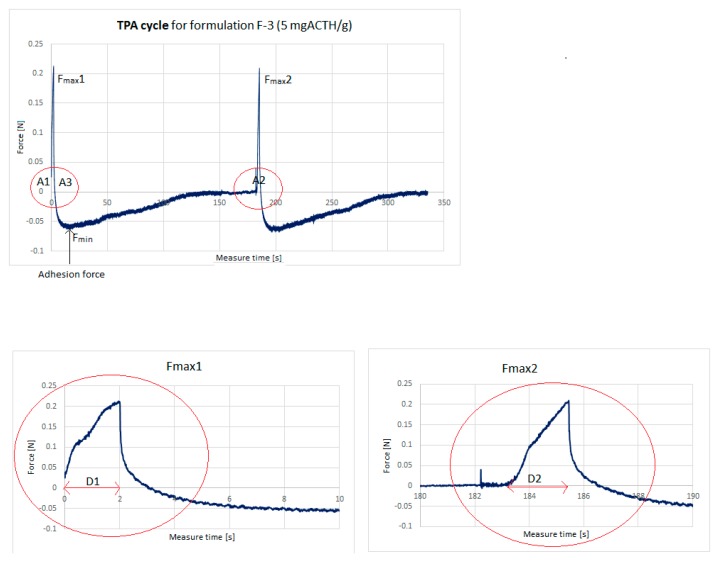
A curve for the texture analysis in the TPA (texture profile analysis) test (one among five for F-3 formulation—5 mg/g ACTH—Adrenocorticotropic hormone).

**Figure 6:**
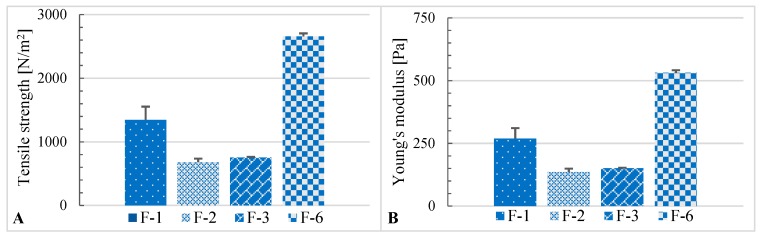
Comparison of: (**A**)—tensile strength profiles for control selected ointments with ACTH (F-3 and F-6) and unloaded bases: F-1 as a F control and F-2 (with 1 mol/L acetic acid solution—10%); (**B**)—Young’s modulus for control selected ointments with ACTH (F-3 and F-6) and unloaded bases: F-1 as a F control and F-2 (with 1 mol/L acetic acid solution—10%).

**Figure 7:**
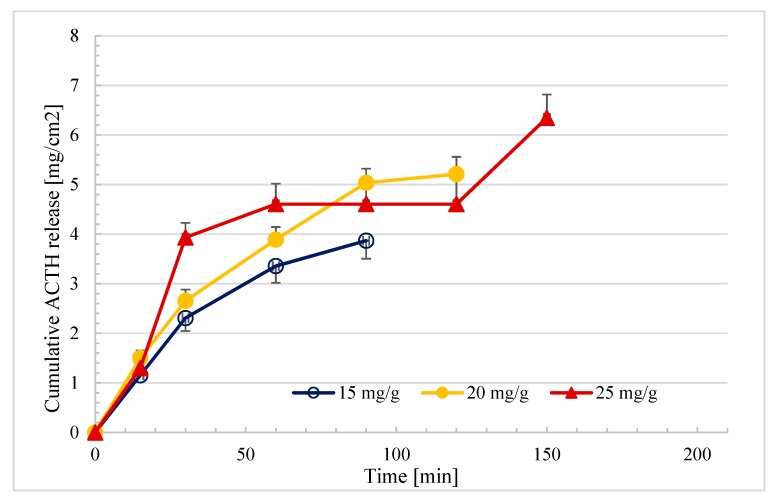
In vitro dissolution profiles of ACTH ointments prepared containing different concentrations of ACTH obtained from a USP 2 apparatus with enhancer cells at 32 °C in water (*n* = 6).

Hysteresis loops of F-2–F-7 at a temperature of 25 °C are shown in Figure 4A-1–4A-7 and the hysteresis loops of F-2–F-7 at a temperature of 32 °C are shown in Figure 4B-1–4B-7. Formulation F-1 is shown as the control ointment. At 32 °C, the hysteresis loops are smaller than at 25 °C, and lower values of their surface were observed. 

Formulations of incompatible compositions tend to produce a structure that is destroyed at high shear rates and which undergoes reforming during ageing at elevated temperatures or excessive pH [35]. It has been reported that the viscosity of hydrogels deteriorates after the gelling agent is roasted and the microcrystalline cellulose (MCC) concentration increases [36]. Moreover, the character of the gelling agent also influences the viscosity parameter: Too high a degree of swelling decreases the degree of swelling and viscosity of the gel formed [37]. In our study, we observed that the addition of an aqueous solution changed the structure of the ointment, similar to the addition of the drug. Perhaps the opposite effect would be due to the addition of more water to the emulsion instead of 10% (e.g., 20%). The smallest changes were caused by 5 and 10 mg/g ACTH.

Based on the hysteresis loops, it can be concluded that, in the case of ointments with a concentration of 5 mg/g (F-3) and Lekobaza^®^ with acetic acid (F-2), the loops overlapped and the ability to recover after deformation was similar for these formulations. The ointment with the highest ACTH content, 25 mg/g, should seem to have the largest surface area, although from the viscosity measurement at three shear rates, it turned out that the ointment with the concentration of 20 mg/g has the highest viscosity, so it can be expected that the formulation at the concentration of 20 mg/g ACTH showed the lowest rheological stability. Based on the significant difference between the initial and final viscosity values during the flow test, which do not overlap at all, the preparation at a concentration of 20 mg/g ACTH can be expected to show the lowest rheological stability. It can be concluded that with the increase of the ACTH content in the ointment, its structure requires more time to return to the original structure: The ascending curve becomes more and more distant from the descending curve. Therefore, the course of viscosity in the range from 100 to 1100 s-1 reflects thixotropy better than single selected shear rates values. The flow test allows the rheological stability of the formulation to be evaluated. 

In comparison, the slope of the non-linear parts of the curves of all preparations at 32 °C is slightly milder than at 25 °C, and the ascending and descending curves are closer together at 32 °C. As the concentration of ACTH increases, the distance between the ascending and descending curves increases. Rheograms received during tests at two temperatures, 25 and 32 °C, show slight changes of the shape of the overall rheogram as the temperature increases. At 32 °C, a decrease in the distance between the ascending and descending curves was observed for each formulation, which is the result of lower shear stress due to the decrease in viscosity at this temperature. Regarding the point of view of the topical application of the preparation, it is a beneficial phenomenon for spreading the preparation on the skin because the temperature of 32 °C corresponds to the temperature on the skin surface.

### 2.4. Texture Analysis

The “TPA Cycle” (texture profile analysis) allows for the performance of a standardized TPA cycle that includes two compression/stretching cycles with a pause between them. These two cycles allow the determination of the hardness, adhesion, elasticity, and cohesion based on the A2/A1 ratio of the two compression phases. Material hardness is defined as the maximum force recorded during the first phase of compression. The value of F_min_ measured in the first phase of stretching is defined as the adhesion force. Adhesion is the work during the first phase. Adhesion is the work needed to overcome the forces of attraction between the ointment surface and the probe surface (work necessary for detachment of the probe from the sample) [38]. The D2/D1 ratio between two compression phase distances is flexible. The values of the marked parameters are presented in Table 4. Four formulations were selected for the study: Lekobaza^®^ (F-1) as a reference, Lekobaza^®^ with an emulsified acetic acid solution (F-2), and two ointment formulations containing the lowest concentration of ACTH (5 mg/g (F-3)) and one of the higher ones of ACTH (20 mg/g (F-6)), to present the effect of the active substance in extreme quantities.

Figure 5 shows an exemplary texture analysis curve of formulation F-3 (5 mg ACTH/g ointment). From the resultant force–time plots, the following mechanical parameters were derived:

- Hardness (the force required to attain a given deformation) was found from maximum force F_max_1.
- Cohesiveness (the ratio of the area under the force–time curve produced on the second compression cycle to that on the first compression cycle, where successive compressions are separated by a defined recovery period).
- Adhesiveness (the work required to overcome the attractive forces between the surface of the sample and the surface of the probe).
- Elasticity: The D2/D1 ratio between two compression phase distances.

Adhesion force: In tension, the force is constantly measured and gives a higher negative value F_min_ and a curve below ‘0′, which characterizes the adhesion of the sample [39].

As shown in Table 4, emulsifying 10% aqueous solution into the medium reduces the hardness by 50%, while the addition of ACTH increases the hardness of the ointment. For example, with ACTH 20 mg/g (F-6), the hardness has doubled in comparison with Lekobaza^®^ (F-1) (*p* < 0.05). The aqueous phase added to the ointment base decreased the adhesion (A) of the ointment by about 1.7 times, but ACTH increases this parameter depending on the amount added, from 1.2 times (5mg/g (F-3)) to more than 5 times (20 mg/g (F-6)) (*p* < 0.05). The adhesion strength (AF) increased with increasing adhesion. As a result, semi-solid formulations can adhere to the application surface for a sufficiently long time, which may affect the drug’s residence time at the application site [40].

When developing preparations for skin and mucous membrane applications, the cohesiveness of the ointment components should be considered [41]. The addition of both water and corticotropin to the tested formulations resulted in decreased cohesiveness. The higher the hormone concentration, the higher the decrease in cohesion (over 3 times (F-6/F-1)) (*p* < 0.05). Similar behaviors were observed in the case of elasticity changes, as water and corticotropin reduced this parameter, with a greater decrease in ACTH 20 mg/g (*p* < 0.05). The more additional ingredients in the ointment, the more it can worsen the texture properties.

Additionally, it is clearly visible that the composition used in the formulation influenced the strength of the designed ointment formulation (Figure 6). The tensile strength and Young’s modulus (modulus of elasticity, [Pa]) were calculated using the Equations (2) and (3):

(2) $$Tensile\;strength=\frac{F}{A},$$

(3) $$Young^{\prime }s\;modulus=\frac{F/A}{\Delta l/l},$$

where *F* [N] represents the force applied to the ointment, *A* [m^2^] displays the calculated cross-sectional area of the ointment cylinder, Δ*l* [m] represents the length deformation, and *l* [m] represents the original sample length. Young’s modulus is a measure of the “stiffness” (mechanical response) of a material, the material’s ability to rebuild its original shape after deformation [42].

With an increasing concentration of ACTH in the ointment, its strength parameters increase. Tensile strength increases, but also the hardness of the ointment increases. With the increase of the textural parameters, the time of release of the active substance may be prolonged. It may indicate the possibility of preparing preparations with a prolonged release process. The addition of an aqueous solution, 1 mol/L acetic acid solution, reduced the tensile strength of the ointment base (F-1) about 2 times. A small addition of ACTH (5 mg/g ointment—F-3) slightly increased the tensile strength of the ointment (F-3), whereas a 4 times higher concentration of ACTH (20 mg/g) caused a twofold increase in the tensile strength of the ointment preparations (F-6) in comparison with F-1 and 4 times in comparison with F-2.

The spreadability of the ointment is inversely proportional to its cohesion, because its internal structure is strong and durable. Cohesive forces reduce its fluidity, and thus its ability to spread on the ground. Therefore, each formulation must be specially designed according to the desired purpose and place of application. The spreadability of the ointment can be increased or decreased, as per the assumption that it meets certain requirements [32,33,43].

### 2.5. In Vitro Drug Release Profiles of the Ointments

A USP apparatus 2 with enhancer cells was utilized to determine the in vitro drug release profiles of the ointment formulations. The results showed that ointments containing different concentrations of ACTH had different rates of release from 90 to 150 min. Figure 7 shows the cumulative amount of released ACTH. ACTH released quicker from the ointments at lower ACTH concentrations (15 and 20 mg/g) and it had a higher drug release rate (90 and 120 min) compared to ointments with a higher ACTH concentration (25 mg/g). In addition, the Higuchi model was employed to calculate the drug release rate of the ACTH ointment formulations (Table 5). In the present study, the time range used for the Higuchi modeling of release rate was 15 to 150 min. The drug release rate and goodness of fit using the Higuchi model for all the ACTH ointment formulations are listed in Table 6. All the formulations showed an adequate fit to the Higuchi model since the R^2^ values are greater than 0.9.

The Weibull model is more useful for comparing the release profiles of matrix-type drug delivery [44]. The Weibull method (*p* < 0.05) showed significant statistical differences in the release profiles of ACTH from prepared ointments of 20 mg/g—F-6 (R^2^ = 0.98) and 25 mg/g—F-7 (R^2^ = 0.81) in relation to the F-5 formulation, from which ACTH was released in the shortest 90 min (R^2^ = 0.97). Based on the t-student analysis, formulations at a concentration of 25 mg/g of ACTH showed significantly lower (*p* < 0.05) drug release rates compared to ointments at concentrations of 20 and 15 mg/g. Based on the Higuchi model, the shortest release time was characteristic for the preparation with the lowest ACTH concentration (15 mg/g-0.41 mg/cm^2^/t^1/2^), ACTH was released from ointments at concentrations of 20 mg/g at a rate of 0.48 mg/cm^2^/t^1/2^, and ACTH was released from ointments at the highest concentration of ACTH for the longest time (20 mg/g-0.52 mg/cm^2^/t^1/2^) (Table 6). The Higuchi model is based on the hypotheses that the initial drug concentration is much higher than the drug solubility, matrix swelling and dissolution are negligible, drug diffusivity is constant, and perfect sink conditions are attained in the release environment [44]. The dependence of the release rate on the ACTH concentration was observed. The higher the concentration, the longer the dissolution and therefore the release time. The release results may be influenced by the nature of the ointment medium, which enables the diffusion process, because in previous studies it was shown that there is no diffusion from such bases of Eucerine or Lekobaza Lux. Among ointment formulations with three different concentrations (15, 20, and 25 mg/g), the ointment with the lowest concentration of ACTH (15 mg/g) in the release test turned out to be the most advantageous, as ACTH was released from this formulation the fastest. The longest ACTH was released from the ointment at a concentration of 25 mg/g and this preparation can provide the effect of prolonged hormone release from the ointment. As far as rheological properties are concerned, the ointment with a concentration of 15 mg/g, from which ACTH was released the fastest, was also characterized by the lowest viscosity in the range of 100–1000 s^−1^, as shown in Table 3 and Figure 4.

The literature reports show that the release process depends on the properties of the membrane through which the therapeutic substance diffuses into the acceptor fluid. It has been reported that artificial cellulose membranes give more reproducible results than natural pork membranes due to the way the natural membrane is prepared. Due to their lipophilic nature, porcine membranes may be more permeable to some substances, whereas artificial ones may be more beneficial to other substances [45,46,47,48,49]. In turn, the use of hairless mouse skin leads to overstated results [50]. The work on synthetic membranes for percutaneous and topical supplies focused on the use of polymeric materials, usually based on silicone. Such membranes are ideal for ex vivo skin replacement as they can be prepared with a specific thickness, are easy to handle and store, are relatively cheap, neutral, and provide reproducible results [51].

The release of ACTH was a preliminary study; therefore, regenerated cellulose membranes were used to optimize the results. Based on the test of release through artificial membranes, it can be preliminarily estimated whether it is possible at all to penetrate large 4.5 kDa particles of ACTH through the skin. In the human skin penetration test, the release process will certainly be slightly different, and this effect can be enhanced by using absorption promoters. The problem of polypeptide permeation through human skin is the next stage of our research.

There are no reports on the penetration of ACTH through the skin in the literature. The idea of administering ACTH as an ointment is a new project, so if you formulate a new form of the drug, it starts with the simplest possible recipe, which can always be improved in the next stages of research development.

The diffusion of a drug molecule depends on many factors: The character of the molecule, its size, drug form, water solubility, oil/water partition coefficient, and physicochemical properties of the drug form. Ideally, when the molecular weight does not exceed 3 kDa or even below 500 Da, log P 1–3, but above 4 are also absorbed [4]. Semi-solid preparations in the form of ointment containing substances, such as heparin, with a particle size from 3000 to 30,000 g/mol, insulin 5809 g/mol, oestradiol—272.38 g/mol, ethinylestradiol—296.40 g/mol, and human gonadotropin—36–46 kDa, are used in the treatment. It is more beneficial when the substance is molecular rather than ionic, as when it is acidic or alkaline rather than saline, lipophilic molecules penetrate skin structures better than hydrophilic ones. However, different forms are used to increase the penetration of molecules through the skin layers, e.g., different absorption promoters can be added, including urea, dimethylsulphoxide (DMSO), and albumin, which modify the release of the therapeutic substance from the dosage form. It is also possible to modify the composition of the ointment medium in order to better release the hormone or the technique of introducing of the active substance into the vehicle [4,52].

## 3. Materials and Methods 

### 3.1. Materials

The research material consisted of ACTH isolated from the pituitary glands, which is easily dissolved in water with activity of 90 IU/mg (Biochefa, Sosnowiec, Poland).

Hydrophilic cream Lekobaza^®^ Fagron (Pharma Cosmetic, Kraków, Poland) with amphiphilic properties was used as a base ointment. To dissolve ACTH before its introduction into the ointment base, 1 mL of 1 mol/L acetic acid solution (POCH, Gliwice, Poland) was used to protect its stability [23].

### 3.2. Preparation of Ointment with Corticotropin

Seven formulations were tested in the study: F-1—Lekobaza^®^, F-2—Lekobaza^®^ with addition 1 mL of acetic acid 1 mol/L, F-3–F-7—ointment with ACTH dissolved in 1 mL acetic acid: F-3—5 mg ACTH per 1 g of ointment, F-4—10 mg ACTH per 1 g ointment, F-5—15 mg ACTH per 1 g ointment, F-6—20 mg ACTH per 1 g ointment, and F-7—25 mg ACTH per 1 g ointment. ACTH was added to Lekobaza after dissolution in acetic acid solution.

Lekobaza is a pure medium—F-1. Lekobaza with acetic acid solution was prepared for comparison with the substrate itself to see what effect the aqueous solution of F-2 has on the physicochemical properties of Lekobaza^®^. Formulations F-3–F-7 are ointments with ACTH. ACTH was introduced into the ointments in the form of an aqueous solution (aqueous acetic acid solution) and emulsified into the ointment base. All ointments with ACTH therefore contain an aqueous solution of acetic acid. If we introduced ACTH directly into Lekobaza^®^, we would have suspended ointments and emulsion-type ointments are characterized by better bioavailability. All ointment formulations were prepared using a Unguator CITO e/s (Eprus, Bielsko-Biała, Poland) prescription mixer. The active substance was emulsified to the base in the form of an aqueous solution. For this purpose, 50.0, 100.0, 150.0, 200.0, and 250.0 mg of corticotropin were weighed and dissolved in 1 mL aqueous acetic acid solution of 1 mol/L. A total of 9 g of ointment base was weighed into a tared 33-mL container and stirred with an Unguator at 5 levels for 2 min (1630 rpm). A corticotropin solution in acetic acid at the specified concentration was added to the prepared ointment base and stirred again at the 5th level rotations for 2 min. Lekobaza^®^ and Lekobaza^®^ with an acetic acid solution of 1 mol/L were homogenized in a Unguator mixer using the same time and speed parameters as the ACTH ointments. Prepared samples were stored at 4 °C. The properties of the emulsion preparations obtained from ACTH were compared with those of the Lekobaza^®^ base. The effect of the addition of 10% aqueous acetic acid to Lekobaza^®^ was also evaluated. The composition of the obtained formulations is presented in Table 7.

**Figure 8:**
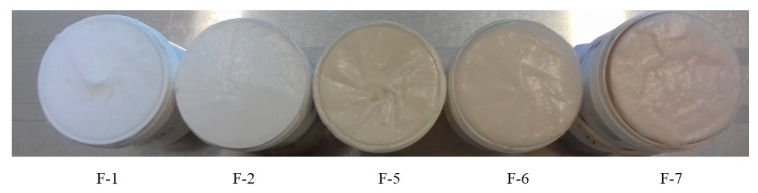
Macroscopic images: ointment base Lekobaza^®^ (F-1), Lekobaza^®^ with acetic acid solution (F-2), Lekobaza^®^ with acetic acid solution and ACTH 15 mg/g of ointment (F-5), Lekobaza^®^ with acetic acid solution and ACTH 20 mg/g of ointment (F-6), and Lekobaza^®^ with acetic acid solution and ACTH 25 mg/g of ointment (F-7).

Figure 8 shows samples of the prepared ointments. The substrate chosen was the same, Lekobaza^®^ and with the addition of acetic acid and three ointments with ACTH at concentrations of 15, 20, and 25 mg/g. All ointments were homogeneous preparations and differed only in color. The more ACTH in the ointment, the more intense the beige pigmentation observed.

### 3.3. pH Measurement

The specification of the drug in the form of dermatological ointment allowed for pH measurement to be performed by the potentiometric method (FP XI)—Polish Pharmacopoeia 11-th edition, 2017 by direct immersion of a glass electrode in a semi-solid dosage form [53]. The electrode In Lab Expert Pro-ISM, Part No. 30014096, Mettler-Toledo AG, Greifensee, Switzerland, was used for the examination. The measurements were taken for the ointment containing respectively: 5 mg/g ACTH, 10 mg/g ACTH, 15 mg/g ACTH, 20 mg/g ACTH, and 25 mg/g ACTH, for Lekobaza^®^ and Lekobaza^®^ with acetic acid solution of 1mol/L at the temperature 25.0 ± 0.5 °C, assumed at the storage or application temperature of the preparations on the skin. Each measurement was carried out five times and the average pH was calculated. pH values are presented as the mean ± standard deviation (SD).

### 3.4. Spreadability

An amount totaling 0.5 cm^3^ of each ointment was placed on a glass plate using a syringe. Then, the second glass plate with a mass of 298.0 g was put at the top without using additional force, and after 30 s, the diameter of the sample was measured. Then, the first weight was placed on the upper plate, and after another 30 s, the diameter of the sample was measured. The procedure was continued gradually from 20.0 to 500.0 g. The test was performed five times for each ointment. The average value of the sample area was calculated. A spreadability test was carried out 24 h after preparation. The dependence of the sample surface of the ointment on the applied load is shown in Figure 1. Table 2 shows the parameters describing the spreadability of the ointment.

### 3.5. Rheological Experiments

Rheological tests were carried out for all ointments with corticotropin (F-3–F-7), Lekobaza^®^ itself, and Lekobaza^®^ with acetic acid solution of 1 mol/L. The analysis was carried out using a universal Reometry RM 200 Touch rotational rheometer by Lamy Rheology Instruments (Champagne au Mont d’Or, France) for testing the rheological properties of the samples. It was equipped with a CP1 Plus thermostat from Lamy Rheology Instruments (Champagne au Mont d’Or, France) with Rheomatic Lamy Instruments software. The tests were carried out on a plate-to-plate geometric system, using the MS CP 2445 Lamy Rheology Instruments (Lamy Rheology, Champagne au Mont d’Or, France) measuring system with a diameter of 24 mm (α = 0.45°). Before measurement, the samples were placed in a CLW 53 STD incubator (POL-EKO Aparatura sp.j., Wodzisław Śląski, Poland) at a temperature of 25.0 ± 0.5 °C and 32.0 ± 0.5 °C.

Before measurement, the samples were placed in a CLW 53 STD incubator (Poland) at a temperature of 25.0 ± 0.5 °C and 32.0 ± 0.5 °C. After 30 min, the sample was placed on the bottom plate of a Peltier effect thermostatic system made of stainless steel, and the upper plate was lowered. The gap between the plates was 50 μm. The excess sample was removed with a spatula.

All rheological tests were carried out at two temperatures: 25.0 ± 0.5 °C and 32.0 ± 0.5 °C.

Two minutes after application of the sample, pre-shear was performed at a shear rate of 100 s^−1^ for 15 s. For the tested ointments, viscosity was measured at three shear rates: 300, 700, and 1100 s^−1^, with shear stress measurement in relation to the shear rate (step by step) and a flow test (flow test). Five repetitions were performed for each experiment, and the mean values of the calculated parameters were presented (*n* = 5).

### 3.6. Texture Analysis

Texture profiles were prepared using the Lamy Rheology Instruments texture analyzer TX-700, (Champagne au Mont d’Or, France). The ointments were in polypropylene containers during the measurements. The texture analysis consisted of compressing the ointment twice with a steel cylindrical sensor (Lamy Rheology, Champagne au Mont d’Or, France), which was immersed in each sample to a depth of 10 mm with a lowering speed of 5 mm/s and an ascent rate of 0.1 mm/s. The initial strength was 0.01 N. The measurements were carried out at 25.0 ± 0.5 °C. The hardness, cohesiveness, adhesiveness, elasticity, and adhesive strength were determined on the basis of the texture profiles (Table 4).

The mechanical properties of the prepared formulations of ointment, including the tensile strength and Young’s modulus, were also conducted.

### 3.7. In Vitro Drug Release from the Ointments

#### 3.7.1. Methodology of ACTH Determination

A standard curve for a corticotropin solution in 0.1 mol/L acetic acid was prepared. The analytical wavelength was determined for spectrophotometric determinations. The spectrum was made in the UV range for a solution of ACTH in 1 mg/mL acetic acid. The wavelength corresponding to the maximum absorbance for ACTH was *λ* = 276.5 nm. Aqueous acetic acid of 0.1 mol/L was used as the reference. Measurements were made using a UV-VIS Cecil CE 3021 spectrophotometer (Cecil Instruments Limited, Cambridge, England).

The calibration curve was based on the absorbance of a corticotropin solution in 0.1 mol/L acetic acid with the following concentrations: 0.1, 0.2, 0.4, 0.5, 0.6, 0.8, and 1.0 mg/mL. Absorbance measurements were taken in 1-cm-thick quartz cuvettes at selected wavelength, *λ* = 276.5 nm. Acetic acid with a concentration of 0.1 mol/L was used as the reference. The arithmetic mean of five samples was calculated. The photometric accuracy of the spectrophotometer was ± 0.005 A. The corticotropin content was calculated from a standard curve with the equation y = 0.5831x − 0.0169; R^2^ = 0.9996.

No interference was found at the selected wavelength chosen *λ* = 276.5 nm for Lekobaza^®^. It was specific only for the ACTH determination.

#### 3.7.2. ACTH Release from Ointment Formulations in Vitro

A modified USP Apparatus 2 equipped with 200-mL flat-bottom vessels and mini paddles (Erweka DT 600, ERWEKA GmbH, Langen, Germany) was used for the drug release studies. An excess amount of the ACTH ointment was loaded inside a special semisolid holder, Enhancer fluoropolymer cells (ERWEKA GmbH, Langen, Germany) with an exposed area of 3.8 cm^2^. Using a spatula, the ointment surface was flattened, smoothed, and excess material removed, after which the exact loaded ointment quantity was determined by weight. As a result, 1.0 g of ointment with the appropriate active substance content was introduced into the Enhancer cells. A pre-cut and pre-wetted regenerated cellulose membrane Spectra/Por^®^2 Dialysis Membrane MWCO: 12–14 kDa, imitating the skin barrier (Fisher Scientific, Loughborough, United Kingdom), was placed on top the ointment before capping the enhancer cell and removing excess water from the membrane. Due to the good solubility of ACTH in water, the release test was conducted in 50 mL of purified water as the acceptor fluid. The process temperature was maintained at 32 ± 0.5 °C throughout the study (it reproduced the temperature of the human skin). The agitation speed of the agitators was 50 rpm. The selected conditions reflected the physiological conditions prevailing in the system. The study was carried out in 150 min. Aliquots of 3 mL were collected at 6 time intervals: 15, 30, 60, 90, 120, and 150 min. The volumes of samples that were taken for measurements were supplemented with 3 mL of purified water at 32 ± 0.5 °C. In samples taken by this method, the amount of released corticotropin was determined by spectrophotometric measurement. On the basis of the obtained results, the amount of released hormone [mg/cm^2^] was calculated per unit of time, and the profiles of its cumulative release were drawn.

### 3.8. Statistical Analysis

Mean values with standard deviation were calculated and statistically analyzed using the Microsoft Excel package (Microsoft, Warsaw, Poland) and Statistica (StatSoft Polska, Cracow, Poland) option: Industrial analysis, experimental design (DOE). The Statistica Pharmaceutical Kit: Statistica “Release Profiles” was used to analyze the results of ACTH release from the ointment bases, with the Weibull method. Statistical significance at *p* < 0.05 was calculated using Student’s t-test.

## 4. Conclusions

An innovative topical ointment formulation of the drug for use on skin containing the hormone corticotropin was obtained and tested. After studies of the physicochemical properties and release, potential benefits were found after both low and high concentrations of ACTH.

The physicochemical properties of the tested ointment formulations are influenced by both the introduction of aqueous solution into the ointment bases and the addition and content of active substance. The addition of aqueous solution improves the spreadability of the ointment and thus enables easy application of the preparation on the skin surface. It allows administration of the ointment in very small quantities (dosages) and reduces systemic toxic effects. Aqueous solution added to the obtained preparations reduces the viscosity, hardness, adhesiveness, elasticity, and mechanical resistance of the ointment to tensile stress. The tested formulations showed smaller thixotropy at a temperature of 32 °C than at 25 °C.

A low concentration of ACTH (5 mg/g) did not significantly affect the texture parameters, while the high concentration of ACTH (20 mg/g) increased the viscosity, hardness, adhesiveness, and tensile strength of the ointment, which in turn adversely affects the cohesiveness and elasticity of the tested ointments and, consequently, makes it difficult to spread the preparation on the skin.

The acetic acid content in the aqueous phase lowers the pH of the ointment, and corticotropin slightly contributes to the increase of the pH value, the higher its concentration in the ointment.

Based on the release test, it can be found that the ACTH release rate depends on its concentration in the ointment. Within the tested concentration range, the release process occurred the fastest from the ointment with the lowest ACTH concentration (15 mg/g), whereas after applying the concentration of 25 mg/g, a prolonged effect can be expected, which may be beneficial in the case of drugs with a short half-life, such as ACTH (5–15 min). The rate of corticotropin release depends on the viscosity and hardness of the ointment. The lower the viscosity and hardness of the ointment formulations, the faster the release of ACTH. As the temperature increases from 25 to 32 °C, the viscosity decreases slightly, which may be beneficial during application of the preparation on the skin.

## Figures and Tables

**Table 1:** The pH values determined for the semi-solid formulations tested (*p* < 0.05).

Formulation	F-1	F-2	F-3	F-4	F-5	F-6	F-7
**pH Value**	6.30 ± 0.02	3.43 ± 0.02	3.61 ± 0.07	3.79 ± 0.04	3.86 ± 0.02	3.92 ± 0.02	4.00 ± 0.02

**Table 2:** Parameters describing the spreadability of the ointments.

Formulation	y=aln(x)+b	R^2^	Surface Area (cm^2^)	i(S)
F-1	20.899*ln(x)* − 92.743	0.943	19,281.4	-
F-2	21.851*ln(x)* − 93.754	0.933	21,786.7	1.130
F-3	19.898*ln(x)* − 82.552	0.931	21,246.4	1.102
F-4	16.791*ln(x)* − 67.144	0.942	19,111.5	0.991
F-5	20.814*ln(x)* − 89.592	0.957	20,523.5	1.064
F-6	20.474*ln(x)* − 86.238	0.937	21,215.6	1.100
F-7	17.752*ln(x)* − 80.112	0.966	15,660.8	0.812

**Table 3:** Viscosity parameters of formulations determined at 25 and 32 °C at three selected shear rates.

Formu-Lation	Shear Rate					
	300 s^−1^		700 s^−1^		1100 s^−1^	
	Viscosity (mPa·s) at 25 °C	Viscosity (mPa·s) at 32 °C	Viscosity (mPa·s) at 25 °C	Viscosity (mPa·s) at 32 °C	Viscosity (mPa·s) at 25 °C	Viscosity (mPa·s) at 32 °C
F-1	1907 ± 43	754 ± 13	1257 ± 26	528 ± 42	741 ± 21	471 ± 8
F-2	1019 ± 74 *	597 ± 7 *	747 ± 25 *	435 ± 2 *	611 ± 16 *	407 ± 12 *
F-3	1102 ± 28 *	584 ± 10 *	738 ± 22 *	423 ± 45 *	596 ± 12 *	397 ± 12 *
F-4	1408 ± 57 *	606 ± 31 *	809 ± 16 *	439 ± 7 *	651 ± 10 *	401 ± 2 *
F-5	1622 ± 13 *	1423 ± 14 *	1096 ± 56 *	984 ± 25 *	877 ± 19 *	644 ± 29 *
F-6	2749 ± 63 *	2272 ± 51 *	1295 ± 137*	1148 ± 10 *	914 ± 44 *	858 ± 13 *
F-7	2071 ± 89 *	1853 ± 32 *	1364 ± 88*	1236 ± 25 *	798 ± 26 *	782 ± 5 *

* Statistically significant difference refers to F-1 (*p* < 0.05).

**Table 4:** Texture parameters of the selected ointments calculated from the texture profile analysis (*p* < 0.05 for F-2, F-3, and F-6) (*n* = 5).

Formulation	Time (s)	Hardness (N)	Cohesiveness	Adhesiveness (mJ)	Elasticity	Adhesion Force F_min_ (N)
F-1	336	0.419 ± 0.070	1.107 ± 0.050	0.567 ± 0.058	1.130 ± 0.026	−0.130 ± 0.003
F-2	335	0.205 ± 0.018	0.885 ± 0.043	0.325 ± 0.050	0.950 ± 0.021	−0.053 ± 0.002
F-3	335	0.227 ± 0.004	0.869 ± 0.036	0.400 ± 0.000	0.947 ± 0.042	−0.059 ± 0.005
F-6	334	0.801 ± 0.015	0.360 ± 0.048	1.683 ± 0.075	0.787 ± 0.166	−0.260 ± 0.012

**Table 5:** Kinetics release models used to describe the release of ACTH from various ointments.

Formulation	Zero Order	First Order	Higuchi Model	Korsmeyer-Peppas Model	Weibull Method
	Regression Coefficient R^2^				
F-5 (15 mg/g)	0.92	0.96	0.98	0.96	0.97
F-6 (20 mg/g)	0.91	0.96	0.98	0.98	0.98
F-7 (25 mg/g)	0.78	0.90	0.92	0.81	0.81

**Table 6:** In vitro drug release rate ang goodness of fit of ACTH ointments using Higuchi’s model (*n* = 6); *—*p* < 0.05.

Formulation	Average Release Rate (mg/cm^2^/min^1/2^) ± SD	R^2^
F-5 (15 mg/g)	0.41 ± 0.02	0.98
F-6 (20 mg/g)	0.48 ± 0.05	0.98
F-7 (25 mg/g)	0.52 ± 0.03 *	0.92

**Table 7:** Composition of the prepared ointment formulations for 10 g of ointment.

	Ingredient	ACTH (mg)	1 mol/L Acetic Acid Solution (mL)	Lekobaza^®^ (g)
Formulation				
F-1		-	-	added to 10.0
F-2		-	1.0	added to 10.0
F-3		50.0	1.0	added to 10.0
F-4		100.0	1.0	added to 10.0
F-5		150.0	1.0	added to 10.0
F-6		200.0	1.0	added to 10.0
F-7		250.0	1.0	added to 10.0
